# Hodgkin Disease—An Ever-Evolving Therapy

**DOI:** 10.5041/RMMJ.10163

**Published:** 2014-10-29

**Authors:** Eldad J. Dann

**Affiliations:** 1 Department of Hematology and Bone Marrow Transplantation, Rambam Health Care Campus, Haifa, Israel;; 2 Blood Bank and Transfusion Service, Rambam Health Care Campus, Haifa, Israel and; 3 Bruce Rappaport Faculty of Medicine, Technion, Israel Institute of Technology, Haifa, Israel

**Keywords:** ABVD, BEACOPP, Hodgkin lymphoma, PET

## Abstract

Therapy of Hodgkin lymphoma (HL) is a rapidly changing field due to plenty of currently emerging data. Treatment approaches are currently based on tailoring of therapy in order to achieve a maximal response with minimal toxicity. Since the median age of HL patients is 33 years and their prospective life expectancy of another half a century, a major emphasis needs to be put on dramatic reduction of later toxicity. The assessment of the treatment effect should be based not only on progression-free survival, but should include evaluation of cardiac toxicity, secondary neoplasms, and fertility in the long-term follow-up. The ancient principle “first do no harm” should be central in HL therapy. Completion of ongoing and currently initiated trials could elucidate multiple issues related to the management of HL patients.

## INTRODUCTION

During the last decade major advances have been made in the treatment of Hodgkin lymphoma (HL). The current review deals with changes in this challenging field, including ongoing and recently completed studies.

The treatment of Hodgkin lymphoma has become one of the most successful stories in oncology. Currently, about 75%–90% of patients can be cured from their disease. Due to this success, long-term toxicity and increased incidence of secondary neoplasms remain major concerns. The median age of HL patients in various studies is 33 years, and patients recovering from their disease have a life expectancy of another half a century, which is an unusual phenomenon in the field of oncology. Major efforts in the last decade concentrated on elucidating both effective and less toxic therapy. Application of positron emission tomography–computed tomography (PET/CT) criteria to differentiate between favorable disease and unfavorable disease, and International Prognostic Score (IPS) used for patients with advanced disease enable the treating physician to determine subgroups of patients with different risks of therapy failure. Currently, the use of PET/CT in Hodgkin lymphoma staging has become the modality of choice, since it was shown to be more accurate than CT, up-staging 19% of patients and down-staging 5% of patients.^1^,^2^ Furthermore, it is replacing bone marrow biopsy during the staging procedure, given that only 1% of patients with negative PET/CT will have bone or bone marrow involvement.^3^–^5^

Interim PET/CT was introduced for risk assessment of the individual patients for treatment failure. At that time, a scan was considered positive “in the presence of a focal concentration of fluorodeoxyglucose (FDG) outside the areas of physiological uptake, with a value increased relative to the background.”^6^ The positive predictive value (PPV) of the study of 260 patients treated with ABVD (doxorubicin, bleomycin, vinblastine, and dacarbazine) was reported to be 84%, with a negative predictive value (NPV) of 95%; the 2-year progression-free survival (PFS) for patients with positive and negative PET-2 was 12.8% and 95%, respectively.^7^ In this study, PET was considered positive if the uptake was above that in the mediastinal blood pool structures and a standardized uptake value was above 3.5. Recently, an international study was published of 260 HL patients treated with ABVD in whom no change of therapy was carried out based on PET/CT.^8^ The 3-year PFS of all patients was 83%, while for those with negative and positive interim PET/CT it was 95% and 28%, respectively. Negative predictive value was 94% and PPV was 73%. These findings led to tailoring of therapy based on interim PET/CT. The Israeli trial was the first study that used interim Ga^67^ imaging, and FDG/PET has been used since 2001 for therapeutic decision-making in patients with advanced disease treated with tailored BEACOPP (bleomycin, etoposide, doxorubicin, cyclophosphamide, vincristine, procarbazine, and prednisone). The initial treatment of the therapeutic arm was defined according to risk factors. Patients with IPS 0–2 started therapy with standard BEACOPP (SB) and those with IPS ≥3 had an initial two cycles of escalated BEACOPP (EB). Following two cycles of therapy, treatment was modified according to interim PET/CT results.^9^ Recently, consensus regarding the interpretation of positive and negative PET/CT was reached using the 5-point scale Deauville score. The PET/CT results interpreted as score 1 (no abnormal uptake) and score 2 (uptake ≤ mediastinal blood pool uptake) are considered negative. Score 4 (uptake moderately above the liver blood pool uptake) and score 5 (uptake markedly above the liver blood pool uptake at any original lesion or appearance of new lesions) are considered positive. Score 3 (uptake above the mediastinal blood pool uptake and ≤ liver blood pool uptake) is interpreted as either positive or negative according to the study design. In studies where, based on PET-2, treatment is de-escalated to less than standard, score 3 is considered positive and therapy is not reduced, while in studies where therapy is escalated based on PET-2, score 3 is considered negative.

This review addresses the following issues that are currently under investigation:

1. Do all patients with early disease benefit from radiation therapy?
2. Should patients with early disease and positive interim PET/CT be treated differently from those with negative interim PET/CT?
3. Should patients with advanced Hodgkin disease and low IPS be treated differently from patients with high IPS?
4. Should all patients receive a more aggressive initial therapy? (studies by the German Hodgkin Study Group (GHSG) and French Lympohoma Study Association (LYSA))
5. Should therapy be escalated based on interim PET/CT imaging performed after two cycles of chemotherapy? (studies by Response-adjusted Therapy for Hodgkin Lymphoma (RATHL), Gruppo Italiano Terapie Innovative nei Linformi (GITIL) HD0607, and Israeli H2)
6. Should treatment be de-escalated according to interim PET/CT results?
7. Should therapy be intensified upfront for all high-risk patients (Israeli H2) or only for those with positive interim PET? (RATHL, GITIL HD0607)
8. Should the treatment protocol incorporate conjugated antibodies? (Echelon study, GHSG)
9. What is the role of radiation therapy in patients with advanced disease?

### 1. DO ALL PATIENTS WITH EARLY DISEASE BENEFIT FROM RADIATION THERAPY?

Radiation therapy was the first effective treatment for Hodgkin lymphoma since the beginning of the twentieth century. The current radiation therapy fields are “involved field“ or “involved nodal,” which are significantly smaller than the previous fields of subtotal nodal irradiation and the cumulative dose is lower, being 20–36 Gy compared to 44 Gy in the past. The Canadian Lymphoma Study Group demonstrated similar 15-year overall survival for patients with early-stage Hodgkin lymphoma treated with or without radiation therapy.^10^

While the PFS was about 3% lower without radiation therapy, the overall survival (OS) was similar. One could deduce from existing data that the incidence of secondary malignancies is expected to rise in about 20 years, so the number of adverse events in the irradiated group is expected to increase. Several studies addressed the question of omitting radiation therapy in early HL, aiming to avoid secondary malignancies. In the study by Pavlovsky et al. from the Argentine Group for Acute Leukemia Treatment (GATLA), 187 patients with early HL stage I or II (non-bulky disease), underwent PET/CT following the third cycle of ABVD.^11^ Seventy percent of these individuals had a negative PET/CT-3 and did not receive any further therapy. The 5-year event-free survival (EFS) in this group was 88%. The RAPID trial in the UK recruited 602 patients with early-stage HL. Altogether 571 patients underwent PET/CT-3 following three cycles of therapy with ABVD (ABVD×3); 74.6% of them had a negative scan (Deauville criteria 1–2) and were randomized for further therapy with or without radiation therapy (RT). The 3-year PFS was 92% for the whole group of negative PET-3, 93.8% in those irradiated using involved field RT (IFRT), and 90.7% in those with no further RT. The overall survival was 97% and 99.5%, respectively.^12^ The largest study to date attempting to evaluate if radiation could be omitted in patients with early disease and negative interim PET post ABVD×2 is the EORTC/LYSA/FIL H10 study. This non-inferiority trial included 1,137 patients, and its experimental arm was prematurely stopped. Patients with favorable HL treated in the standard arm received involved nodal radiation therapy (INRT) of 30 Gy following ABVD×2. In this arm there was a single event; however, in the experimental arm, where patients did not receive RT nine events were reported. As a result, the 1-year PFS in the two arms was 100% and 94.9%, respectively. For patients in the early unfavorable group, seven events were registered in the standard arm and 16 events in the experimental arm; hence, the 1-year PFS was 97.3% and 94.7%, respectively.^13^ One may conclude that omission of radiation therapy is accompanied with about 3%–4% reduction in PFS. However, the majority of patients may be salvaged later with radiation therapy, and a long-term follow-up of 20 years or more is needed to elucidate if the radiation arm provides benefit. The data from the Canadian Lymphoma Study Group support omitting radiation therapy due to a better long-term overall survival.^10^ Patients enrolled in the Israeli Study Group who had a negative interim PET/CT had the possibility to choose between further chemotherapy and radiation therapy. The majority of patients elected not to have radiation therapy in order to reduce long-term toxicity.

### 2. SHOULD PATIENTS WITH EARLY DISEASE AND POSITIVE INTERIM PET/CT BE TREATED DIFFERENTLY FROM THOSE WITH NEGATIVE INTERIM PET/CT?

This issue is being currently investigated by at least six study groups in various trials. Early studies evaluating the prognostic value of interim PET/CT have been conducted at Guy’s and St Thomas’ Hospital in London since 1993. Eighty-five patients (57 of them with early disease) underwent PET/CT following two (55 patients) or three cycles (30 patients) of therapy. Of 63 patients with a negative scan, three progressed. Nine patients had minimal residual uptake, and one progressed. Two of seven patients with early disease and positive interim PET relapsed. The 5-year PFS in patients with negative and positive interim PET was 91.5% and 38.5%, respectively.^14^ The EORTC/LYSA/FIL H10 study includes 60 patients with early favorable disease and 164 patients with early unfavorable disease who had positive interim PET-2. In about half of these 224 patients therapy was intensified with EB×2 followed by INRT, since they were included in the experimental arm. Data regarding the outcome of patients in these groups are not yet published but would be very valuable when available. Primary results of the RAPID trial in the UK for patients with early-stage non-bulky disease demonstrated in 145 patients with positive interim PET/CT, who were treated with the addition of a fourth ABVD cycle following the PET/CT scan and IFRT, a 3-year PFS and OS of 85.9% and 93.9%, respectively, while 209 patients with a negative interim scan who were treated with ABVD×3 and IFRT had a 3-year PFS of 93.8% and OS of 97%.^12^ Ninety-nine patients with early Hodgkin lymphoma were treated with gemcitabine, adriamycin, and vinblastine in the CALGB 50203 study, where PET-2 was performed. The 3-year PFS for the whole group was 77% (95% CI, 68%–84%). The 2-year PFS for PET-2-negative and -positive patients was 88% and 54%, respectively (*P*=0.0009).^15^

Patients treated in the Argentine GATLA Group study who had a positive PET-3 had a 4-year event-free survival (EFS) of 55%, while patients with negative PET-3 had EFS of 88%.

Preliminary data from the Israeli H2 study demonstrated a hazard ratio for relapse of 2.8 (1–7.9; *P*=0.06) for patients with positive interim PET/CT compared to those with negative interim scan. These results demonstrate that moderate escalation of therapy with additional ABVD×2 and IFRT could not overcome the inferior prognosis associated with positive interim scan.

The final results of the currently ongoing studies may elucidate these two issues related to confirmation of inferior prognosis for patients with positive interim PET/CT and possibility to ameliorate significantly this adverse prognostic sign with therapy escalation.

### 3. SHOULD PATIENTS WITH ADVANCED HODGKIN DISEASE AND LOW IPS BE TREATED DIFFERENTLY FROM PATIENTS WITH HIGH IPS?

The IPS was introduced in 1998 by Hasenclever et al.^16^ demonstrating a 7%–10% inferiority of freedom from progression (FFP) with addition of each point in the IPS. While patients with IPS=0 had a 5-year FFP of 84%, in patients with IPS 5–7 this parameter amounted to 42% only. A recent retrospective study of 740 patients conducted by the Hodgkin Lymphoma Registry in British Columbia revealed a narrower distribution between the high and low IPS; however, differences in the 5-year FFP ranging between 62% for patients with IPS>4 and 88% for patients with IPS=0 (*P<*0.001) and in the 5-year OS ranging between 67% and 98% (*P<*0.001) were still highly significant.^17^ In a recent Eastern Cooperative Oncology Group study (ECOG 24963) of patients with advanced disease treated with either ABVD or Stanford V, the 5-year FFP was 82%, 74%, 63% for patients with IPS 0–2, 3–4, 5–7, respectively.^18^ Federico et al. demonstrated an inferior outcome for patients with IPS 3–7 compared to those with IPS 0–2 in the GISL HD2000 study.^19^ These studies led to the following recommendation in the latest guidelines for Hodgkin lymphoma therapy: “Patients with a higher IPS are at higher risk of relapse,” potentially supporting the use of EB in this higher-risk group, although there are no prospective trial data to confirm a specific IPS cut-off at which EB may be advantageous.^20^ The Israeli H2 study had a cut-off point for starting treatment of patients with a low IPS 0–2 with ABVD and patients with IPS 3–7 with EB. Preliminary results show no difference in PFS between patients with low and high scores.^21^

### 4. SHOULD ALL PATIENTS RECEIVE A MORE AGGRESSIVE INITIAL THERAPY?

There has been a hot ongoing debate on this issue since 1998 when the first results of the GHSG HD9 study were presented, followed by final results of this study.^22^ While the findings demonstrated a better PFS for escalated therapy, patients suffered the consequences of impaired fertility (in more than 50% of females) and an increased rate of secondary leukemias. Furthermore, about 70% of the patients in this trial received RT, a modality that could lead to a high rate of secondary tumors at long-term follow-up. A later HD15 study of the GHSG^23^ recently claimed that EB×6 should be the current standard of care since the reported 5-year OS was 95%. Skoetz et al.^24^ concluded that EB×6 or BEACOPP14×8 is a new standard of care. This claim was based on a meta-analysis that included some old and new studies demonstrating an 88% 5-year survival for ABVD trials and a 95% 5-year OS for the HD15 study that used EB×6. On the other hand, investigators that favored the less toxic approach of initial ABVD use^25^ argued that recent results of the European Organization for Research and Treatment of Cancer (EORTC) 20012 study showed a 5-year OS of 90%,^26^ and this parameter in the GISL HD2000 trial amounted to 92%,^19^ which probably makes the difference in OS minimal. A head-to-head comparison carried out by Federico et al. in the HD2000 study showed a 5-year PFS for the ABVD×6 versus EB×4+SB×2 of 68% and 81%, respectively, and OS of 84% and 92%, respectively.^19^ In another head-to-head comparison conducted by Viviani et al.,^27^ the 7-year PFS was 85% versus 73% for the arm receiving EB×4+SB×4 versus the arm treated with ABVD×6 (*P*=0.004). The freedom from second progression was 88% and 82%, respectively (NS), and no significant differences in OS were demonstrated (89% and 84%). In the Israeli tailored BEACOPP study that was based on both IPS and interim PET/CT, only six cycles of therapy were given, and the majority of patients received EB×2 and SB×4. The 10-year PFS and OS were 87% and 88%, respectively, with no difference in the patients with IPS 0–2 upfront treated with SB and patients with IPS 3–7 upfront treated with EB, suggesting that not all patients should be treated aggressively upfront. In the last-mentioned study, 94% of women preserved their cyclic ovarian function; 30 spontaneous pregnancies and 24 successful deliveries were reported in this group, demonstrating some merit for minimizing the use of aggressive therapy.^28^

In some currently ongoing studies, PET/CT-2 is performed following two cycles of therapy with EB in the control arm, but treatment is not changed based on the scan results, while in the experimental arm treatment is de-escalated based on negative PET/CT-2 to either ABVD×4 as in the LYSA AHL 2011 study or to EB×2 as in GHSG HD18.^29^ It is not yet elucidated if there is benefit in upfront starting with more aggressive therapy for all patients (kairos principle) and reducing therapy based on negative interim PET, or in using ABVD upfront with therapy escalation only for patients with positive interim PET/CT (chronos principle). The chronos approach was recently studied in the RATHL study that recruited 1,214 patients with advanced disease. All patients initiated treatment with ABVD, and 84% of them had a negative interim PET/CT. Sixteen percent of patients had a positive interim PET-2, and their therapy was escalated to either EB×4 or BEACOPP14×6. Only 25% of the patients with positive interim PET had a positive PET at the end of therapy. At 18-month follow-up, the PFS for the whole cohort did not differ for patients with IPS 0–2 compared to those with IPS 3–7; however, a longer follow-up is needed to draw conclusions.

The Israeli H2 protocol is designed so that only patients with a high IPS are treated upfront with EB×2 due to their higher risk to have positive PET/CT-2 following two cycles of therapy; however, in 80% of these patients PET/CT-2 was negative, and their therapy was de-escalated in order to minimize toxicity.

The available data suggest that not all patients benefit from aggressive first-line therapy, and once the data mature it will be possible to compare the results of the two schools of thought. Unfortunately, to date there is only a single randomized prospective study comparing these two treatment concepts. In the control arm of this trial, early interim PET/CT is performed following EB×1 with no change in treatment based on PET/CT-2 (a total of EB×6). In the experimental arm, PET-2 is performed following ABVD×1, and patients with a negative interim scan are treated with ABVD×6, while those with a positive interim scan have their therapy escalated to EB (EORTC H11).

### 5. SHOULD THERAPY BE ESCALATED BASED ON INTERIM PET/CT IMAGING PERFORMED AFTER TWO CYCLES OF CHEMOTHERAPY? (RATHL, GITIL, ISRAELI H2)

The latest guidelines for therapy of Hodgkin lymphoma acknowledge that the optimal management of patients with positive interim PET/CT remains uncertain. “Hence, at this time, interim PET/CT is desirable for patients treated with ABVD, but it cannot be mandated as standard of care.”^20^ There is ample evidence demonstrating that patients with a positive interim PET/CT following ABVD therapy have an inferior PFS. In a retrospective international multicenter study, negative predictive value of interim PET-2 was around 95%, and PFS of patients with positive interim PET was 28%.^8^ Progression-free survival was only 13% in an earlier study which analyzed results of the Italian-Danish groups.^7^

In patients with advanced disease treated with ABVD upfront in whom therapy was escalated based on interim PET-2, a failure-free survival (FFS) of 65% was reported. The FFS for patients with negative interim PET was 92%.^30^ Preliminary data from prospective studies like RATHL demonstrate that 16% of patients with advanced disease initially treated with ABVD×2 have positive interim PET-2. In this subgroup, therapy escalation provided a complete response at the end of chemotherapy (Deauville score 1–3) in 76% of patients.^31^ In the Italian GITIL HD0607 for patients with HD IPS 0–7, the whole cohort was initially treated with ABVD. Following two cycles of therapy, PET-2 was negative in 85% of patients. In patients with positive PET-2, therapy was escalated to EB or rituximab+EB. The 2-year FFS was 67%.

In conclusion, 80%–85% of patients treated with ABVD×2 upfront have a negative interim PET/CT, and 15%–20% have a positive scan defined as an uptake higher than in the liver (Deauville 4). Escalation of therapy could salvage 60%–76% of these patients.^30^,^31^ In my opinion, these data provide solid evidence both for mandatory performance of interim PET/CT following ABVD×2 and for escalation of therapy if PET-2 is positive.

### 6. SHOULD TREATMENT BE DE-ESCALATED ACCORDING TO INTERIM PET/CT RESULTS?

While a more intensive therapy like EB may increase the percentage of patients with high IPS who have negative PET-2, the issue of a higher toxicity prevents many physicians from adopting the kairos principle. Two phase II studies that have already matured used this approach.

The Israeli tailored BEACOPP study reported a 10-year PFS of 87% and OS of 87.7% for 47 patients with IPS 3–7 who had their treatment reduced after EB×2 to a further SB×4 based on negative interim PET/CT.^28^ In another Israeli study, 31 patients with IPS 3–7 in whom treatment was changed following EB×2 to a further four cycles of standard ABVD, based on negative interim PET/CT, had a 4-year PFS of 87%.^32^ In the H2 study a subgroup of 69 patients with IPS ≥3 initially received EB×2. Eighty percent of these patients had a negative interim PET/CT-2, and their therapy was de-escalated to ABVD×4. Only 10% of these patients relapsed, with a 3-year PFS of 84% for the whole group.^21^ Similarly, the GHSG HD18 is currently evaluating the possibility to reduce therapy for patients initially treated with EB×2 who had negative interim PET-2. Patients randomized to the control arm receive four additional cycles of EB (total of six cycles), while patients in the experimental arm are treated with two additional cycles of EB only (total of four cycles of EB) if their interim PET is negative.^29^ The GELA AHL2011 study presently randomizes patients with advanced HD (IPS 0–7) who are initially treated with EB×2. Patients in the control arm receive EB×6 with no change in therapy based on PET-2 results. Patients in the experimental arm have their therapy de-escalated to ABVD×4 if PET-2 is negative. Patients with a positive PET-2 continue with EB×4. It is expected that this currently ongoing randomized trial will provide quality data regarding the possibility of therapy de-escalation if EB is initiated according to the kairos principle.

### 7. SHOULD THERAPY BE INTENSIFIED UPFRONT FOR ALL HIGH-RISK PATIENTS (ISRAELI H2) OR ONLY FOR THOSE WITH POSITIVE INTERIM PET? (RATHL, GITIL)

The chronos philosophy of treatment is “first to do no harm,” and thus only patients with a clear high risk for disease progression indicated by positive PET-2 should have their treatment intensified to EB or in some studies (like FIL 0801) even to salvage therapy with autologous stem cell transplantation.^33^ However, when electing to start with ABVD for patients with IPS 0–7, one should bear in mind that the available data demonstrate that the percentage of patients who will have negative PET-2 following ABVD×2 is inversely correlated to their IPS. The study of 260 patients showed that positive interim PET was a marker of a poor prognosis; while only 12.8% of patients with IPS 0–2 had a positive interim scan, this value increased to 38.5% in patients with IPS ≥3.^7^ An international study examined the prognostic predictive value of another 260 patients according to the Deauville scoring system. In this cohort, 12% of patients with IPS 0–2 and 30% of patients with IPS ≥3 had a positive interim scan. Further data regarding this issue will be available when the data from the RATHL and GITIL studies are published. Both these studies initiate treatment of all patients (IPS 1–7) with ABVD. In the Israeli H2 study, patients with IPS ≥3 start therapy with EB, while patients with IPS 0–2 are initially treated with ABVD. In the cohort of patients (IPS ≥3) treated with EB×2, 81% of individuals had negative interim PET compared to 62% of patients with IPS ≥3 initially treated with ABVD×2 in the international validation study.^8^,^21^ These findings suggest that for patients with a higher IPS the probability of a negative interim scan with an initial aggressive therapy of EB×2 is better than with ABVD×2.

### 8. SHOULD TREATMENT PROTOCOLS INCORPORATE CONJUGATED ANTIBODIES? (ECHELON STUDY, GHSG)

To date, there are no mature data to answer this question. Brentuximab vedotin is an anti-CD30 antibody that is linked to monomethyl auristatin E (MMAE), a potent antitubulin agent. Once the antibody is attached to a CD30-positive cell the antibody is engulfed and the protease cleavable linker is degraded. Then, the antitubulin agent becomes active and damages the cell. In a phase II study^34^ approximately 75% of patients with refractory disease had response to the antibody and one-third of patients achieved complete remission. Grade I–II neurotoxicity was reported in 30% of patients. In a cohort of heavily pretreated patients, four individuals developed progressive multifocal leukoencephalopathy.

Brentuximab vedotin was shown to be highly toxic when combined with bleomycin. Currently, an international study is comparing the upfront use of A2VD (adriamycin, brentuximab vedotin, vinblastine, and dacarbazine) versus ABVD for patients with advanced HL disease. Following two cycles of therapy, interim PET-2 is carried out. Only patients with Deauville score 5, denoting disease progresssion, are going off-study. It is expected that the study will answer the question whether combined therapy is advantageous for patients with negative interim PET-2 and, if so, at what cost in terms of toxicity. It might also show if a higher percentage of patients have a negative interim PET-2, and if the negative predictive value of negative interim scan in patients treated with A2VD provides a prognosis similar to that of patients treated with ABVD. A different approach was used by the GHSG comparing two regimens which combined chemotherapy with brentuximab vedotin. This new trial aims to compare the efficacy of EB×6 given as first-line therapy with that of a modified protocol using brentuximab vedotin antibodies while omitting procarbazine and bleomycin in the BrECADD protocol (brentuximab vedotin, etoposide, cyclophosphamide, adriamycin, dacarbazine, dexamethasone). It will probably take a few years until we learn the role of the conjugated antibody brentuximab vedotin in first-line therapy.

### 9. WHAT IS THE ROLE OF RADIATION THERAPY IN PATIENTS WITH ADVANCED DISEASE?

The use of radiation therapy following first-line treatment of advanced HL changed dramatically once PET/CT was introduced. In the GHSG HD9 study which claimed that EB×8 should become the standard of care for advanced HL, about 70% of patients received radiation therapy. The GHSG HD12 enrolled 1,670 patients with HL stage III or IV who were randomized to be given BEACOPP esc ×8 or BEACOPP (esc ×4 +base ×4).^35^ The 5-year freedom from treatment failure (FFTF) was inferior in the arm that did not receive RT (90.4% versus 87%; difference −3.4%; 95% CI −6.6% to −0.1%), particularly in patients who had residual disease after chemotherapy (−5.8%), but not in patients with a bulky disease who had a complete response after chemotherapy. In the GHSG HD15 which claimed that EB×6 should become the standard of care for advanced HL, about 11% of patients who had a persistent mass (measuring 2.5 cm or more) after chemotherapy and a positive PET scan received additional radiotherapy with 30 Gy. The currently ongoing HD18 study is also designed so that only patients with a positive PET at the end of therapy should undergo RT. The conclusion from these studies is that patients with negative PET and a residual mass have the same FFS without exposure to RT. Is this also true for patients treated upfront with ABVD? Recently published studies, by the UK National Cancer Research Institute (NCRI), ISRCTN 64141244,^36^ Intergruppo Italiano Linfomi,^28^,^37^ Eastern Cooperative Oncology Group (ECOG),^18^ and Gruppo Italiano Per Lo Studio Dei Linfomi HD 2000,^19^ all had radiation therapy included in the treatment regimen of patients with a residual mass. Radiation therapy was administered to 41%–66% of the patients. While other protocols, employed by the European Organization for Research and Treatment of Cancer (EORTC) 20012^26^ and Lymphoma Study Association (LYSA) H34,^38^ did not include RT, their PFS was similar to that observed in the studies that had RT as part of the treatment regimens. In the UK NCRI Lymphoma Study Group RATHL trial which was designed as a non-inferiority study, two subgroups of patients were not scheduled to receive RT: one subgroup included patients with negative PET-2, and the other subgroup incorporated individuals with positive PET-2 in whom PET-3 was negative following EB×3. Less than 5% of patients with Deauville score 1–3 underwent RT.^31^ These study results when mature will most probably support the decision to omit radiation therapy for patients with negative interim PET. In the ongoing Israeli H2 study, where patients with negative interim PET do not receive RT if the end-of-therapy scan is negative, preliminary data do not support the need for RT in this patient population.

## CONCLUSION

In conclusion, currently ongoing studies may supply data that would change the therapeutic approach to the management of HL based on differentiation between low-risk patients in whom less toxic therapy could be employed and high-risk patients in whom a more intensive therapy is required.

## Abbreviations:

ABVD: doxorubicin, bleomycin, vinblastine, dacarbazine;
BEACOPP: bleomycin, etoposide, doxorubicin, cyclophosphamide, vincristine, procarbazine, prednisone;
EB: escalated BEACOPP;
EFS: event-free survival;
FDG: fluorodeoxyglucose;
GATLA: Group for Acute Leukemia Treatment;
GHSG: German Hodgkin Study Group;
GITIL: Gruppo Italiano Terapie Innovative nei Linformi;
HL: Hodgkin lymphoma;
INRT: involved nodal radiation therapy;
IPS: International Prognostic Score;
LYSA: French Lympohoma Study Association;
NPV: negative predictive value;
PET/CT: positron emission tomography–computed tomography;
PPV: positive predictive value;
RATHL: Response-adjusted Therapy for Hodgkin Lymphoma;
RT: radiation therapy;
SB: standard BEACOPP.

## References

[1] Hutchings M, Loft A, Hansen M (2006). Position emission tomography with or without computed tomography in the primary staging of Hodgkin’s lymphoma. Haematologica.

[2] Raanani P, Shasha Y, Perry C (2006). Is CT scan still necessary for staging in Hodgkin and non-Hodgkin lymphoma patients in the PET/CT era?. Ann Oncol.

[3] El-Galaly TC, d’Amore F, Mylam KJ (2012). Routine bone marrow biopsy has little or no therapeutic consequence for positron emission tomography/computed tomography-staged treatment-naive patients with Hodgkin lymphoma. J Clin Oncol.

[4] Barrington SF, Mikhaeel NG, Kostakoglu L (2014). Role of imaging in the staging and response assessment of lymphoma: consensus of the International Conference on Malignant Lymphomas Imaging Working Group. J Clin Oncol.

[5] Cheson BD, Fisher RI, Barrington SF (2014). Recommendations for initial evaluation, staging, and response assessment of Hodgkin and non-Hodgkin lymphoma: the Lugano Classification. J Clin Oncol.

[6] Gallamini A, Rigacci L, Merli F (2006). The predictive value of positron emission tomography scanning performed after two courses of standard therapy on treatment outcome in advanced stage Hodgkin’s disease. Haematologica.

[7] Gallamini A, Hutchings M, Rigacci L (2007). Early interim 2-[18F]fluoro-2-deoxy-D-glucose positron emission tomography is prognostically superior to international prognostic score in advanced-stage Hodgkin’s lymphoma: a report from a joint Italian-Danish study. J Clin Oncol.

[8] Gallamini A, Barrington SF, Biggi A (2014). The predictive role of interim positron emission tomography for Hodgkin lymphoma treatment outcome is confirmed using the interpretation criteria of the Deauville five-point scale. Haematologica.

[9] Dann EJ, Bar-Shalom R, Tamir A (2007). Risk-adapted BEACOPP regimen can reduce the cumulative dose of chemotherapy for standard and high-risk Hodgkin lymphoma with no impairment of outcome. Blood.

[10] Meyer RM, Gospodarowicz MK, Connors JM (2012). ABVD alone versus radiation-based therapy in limited-stage Hodgkin’s lymphoma. N Engl J Med.

[11] Pavlovsky A, Fernandez I, Zoppegno L (2013). PET-CT adapted therapy after three cycles of ABVD to all stages of Hodgkin lymphoma: a report from the GATLA Group. Hematol Oncol.

[12] Radford J, Barrington S, Counsell N (2012). Involved field radiotherapy versus no further treatment in patients with clinical stages IA and IIA Hodgkin lymphoma and a ‘negative’ PET scan after 3 cycles. ABVD Results of the UK NCRI RAPID Trial. Blood.

[13] Dann EJ, Bairey O, Bar-Shalom R (2013). Tailored therapy in Hodgkin lymphoma, based on predefined risk factors and early interim PET/CT, Israeli H2 protocol: preliminary report on 317 patients. Haematologica.

[14] Carde PP, Karrasch M, Fortpied C (2012). ABVD (8 cycles) versus BEACOPP (4 escalated cycles => 4 baseline) in stage III–IV high-risk Hodgkin lymphoma (HL): first results of EORTC 20012 Intergroup randomized phase III clinical trial. J Clin Oncol.

[15] Johnson P, Federico M, Fossa A (2013). Response rates and toxicity of response-adapted therapy in advanced Hodgkin lymphoma: initial results from the International RATHL study. Haematologica.

[16] Raemaekers JM, Andre MP, Federico M (2014). Omitting radiotherapy in early positron emission tomography-negative stage I/II Hodgkin lymphoma is associated with an increased risk of early relapse: clinical results of the preplanned interim analysis of the randomized EORTC/LYSA/FIL H10 trial. J Clin Oncol.

[17] Hutchings M, Mikhaeel NG, Fields PA (2005). Prognostic value of interim FDG-PET after two or three cycles of chemotherapy in Hodgkin lymphoma. Ann Oncol.

[18] Straus DJ, Johnson JL, LaCasce AS (2011). Doxorubicin, vinblastine, and gemcitabine (CALGB 50203) for stage I/II nonbulky Hodgkin lymphoma: pretreatment prognostic factors and interim PET. Blood.

[19] Hasenclever D, Diehl V (1998). A prognostic score for advanced Hodgkin’s disease. International Prognostic Factors Project on Advanced Hodgkin’s Disease. N Engl J Med.

[20] Moccia AA, Donaldson J, Chhanabhai M (2012). International Prognostic Score in advanced-stage Hodgkin’s lymphoma: altered utility in the modern era. J Clin Oncol.

[21] Gordon LI, Hong F, Fisher RI (2013). Randomized phase III trial of ABVD versus Stanford V with or without radiation therapy in locally extensive and advanced-stage Hodgkin lymphoma: an intergroup study coordinated by the Eastern Cooperative Oncology Group (E2496). J Clin Oncol.

[22] Federico M, Luminari S, Iannitto E (2009). ABVD compared with BEACOPP compared with CEC for the initial treatment of patients with advanced Hodgkin’s lymphoma: results from the HD2000 Gruppo Italiano per lo Studio dei Linfomi Trial. J Clin Oncol.

[23] Follows GA, Ardeshna KM, Barrington SF (2014). Guidelines for the first line management of classical Hodgkin lymphoma. Br J Haematol.

[24] Diehl V, Franklin J, Pfreundschuh M (2003). Standard and increased-dose BEACOPP chemotherapy compared with COPP-ABVD for advanced Hodgkin’s disease. N Engl J Med.

[25] Engert A, Haverkamp H, Kobe C (2012). Reduced-intensity chemotherapy and PET-guided radiotherapy in patients with advanced stage Hodgkin’s lymphoma (HD15 trial): a randomised, open-label, phase 3 non-inferiority trial. Lancet.

[26] Skoetz N, Trelle S, Rancea M (2013). Effect of initial treatment strategy on survival of patients with advanced-stage Hodgkin’s lymphoma: a systematic review and network meta-analysis. Lancet Oncol.

[27] Federico M, Luminari S, Dondi A (2013). R-CVP versus R-CHOP versus R-FM for the initial treatment of patients with advanced-stage follicular lymphoma: results of the FOLL05 Trial conducted by the Fondazione Italiana Linfomi. J Clin Oncol.

[28] Viviani S, Zinzani PL, Rambaldi A (2011). ABVD versus BEACOPP for Hodgkin’s lymphoma when high-dose salvage is planned. N Engl J Med.

[29] Dann EJ, Blumenfeld Z, Bar-Shalom R (2012). A 10-year experience with treatment of high and standard risk Hodgkin disease: six cycles of tailored BEACOPP, with interim scintigraphy, are effective and female fertility is preserved. Am J Hematol.

[30] Borchmann P, Eichenauer DA, Engert A (2012). State of the art in the treatment of Hodgkin lymphoma. Nat Rev Clin Oncol.

[31] Gallamini A, Patti C, Viviani S (2011). Early chemotherapy intensification with BEACOPP in advanced-stage Hodgkin lymphoma patients with a interim-PET positive after two ABVD courses. Br J Haematol.

[32] Avigdor A, Bulvik S, Levi I (2010). Two cycles of escalated BEACOPP followed by four cycles of ABVD utilizing early-interim PET/CT scan is an effective regimen for advanced high-risk Hodgkin’s lymphoma. Ann Oncol.

[33] Zinzani PL, Bonfichi M, Rossi G (2013). Early salvage with high-dose chemotherapy and stem cell transplantation in advanced stage Hodgkin’s lymphoma patients with positive positron emission tomography after two courses of chemotherapy: preliminary results of the IIL-HD0801 study. Haematologica.

[34] Younes A, Gopal AK, Smith SE (2012). Results of a pivotal phase II study of brentuximab vedotin for patients with relapsed or refractory Hodgkin’s lymphoma. J Clin Oncol.

[35] Borchmann P, Haverkamp H, Diehl V (2011). Eight cycles of escalated-dose BEACOPP compared with four cycles of escalated-dose BEACOPP followed by four cycles of baseline-dose BEACOPP with or without radiotherapy in patients with advanced-stage Hodgkin’s lymphoma: final analysis of the HD12 trial of the German Hodgkin Study Group. J Clin Oncol.

[36] Hutchings M (2012). How does PET/CT help in selecting therapy for patients with Hodgkin lymphoma?. Hematology Am Soc Hematol Educ Program.

[37] Gobbi PG, Levis A, Chisesi T (2005). ABVD versus modified Stanford V versus MOPPEBVCAD with optional and limited radiotherapy in intermediate- and advanced-stage Hodgkin’s lymphoma: final results of a multicenter randomized trial by the Intergruppo Italiano Linfomi. J Clin Oncol.

[38] Mounier N, Brice P, Bologna S (2013). Standard ABVD vs. escalated BEACOPP in stage III – IV low risk Hodgkin lymphoma (IPS 0-2): the Lymphoma Study Association (LYSA) H34 trial. Hematol Oncol.

